# Systems biology of cancer: moving toward the integrative study of the metabolic alterations in cancer cells

**DOI:** 10.3389/fphys.2012.00481

**Published:** 2013-01-03

**Authors:** Claudia E. Hernández Patiño, Gustavo Jaime-Muñoz, Osbaldo Resendis-Antonio

**Affiliations:** ^1^Undergraduate Program for Genomic Sciences, Universidad Nacional Autónoma de MéxicoCuernavaca, México; ^2^Biomedical Science Posgraduated Program, Universidad Nacional Autonoma de MexicoMéxico, México; ^3^Cancer Systems Biology Group, Instituto Nacional de Medicina Genómica (INMEGEN)México, México

**Keywords:** computational modeling of metabolism, cancer metabolic phenotype, constraint-based modeling, genome scale metabolic reconstruction, high throughput biology

## Abstract

One of the main objectives in systems biology is to understand the biological mechanisms that give rise to the phenotype of a microorganism by using high-throughput technologies (HTs) and genome-scale mathematical modeling. The computational modeling of genome-scale metabolic reconstructions is one systemic and quantitative strategy for characterizing the metabolic phenotype associated with human diseases and potentially for designing drugs with optimal clinical effects. The purpose of this short review is to describe how computational modeling, including the specific case of constraint-based modeling, can be used to explore, characterize, and predict the metabolic capacities that distinguish the metabolic phenotype of cancer cell lines. As we show herein, this computational framework is far from a pure theoretical description, and to ensure proper biological interpretation, it is necessary to integrate high-throughput data and generate predictions for later experimental assessment. Hence, genome-scale modeling serves as a platform for the following: (1) the integration of data from HTs, (2) the assessment of how metabolic activity is related to phenotype in cancer cell lines, and (3) the design of new experiments to evaluate the outcomes of the *in silico* analysis. By combining the functions described above, we show that computational modeling is a useful methodology to construct an integrative, systemic, and quantitative scheme for understanding the metabolic profiles of cancer cell lines, a first step to determine the metabolic mechanism by which cancer cells maintain and support their malignant phenotype in human tissues.

## Introduction

Cancer is a complex disease that is characterized by uncontrolled cell growth. At the genetic level, cancer research has focused primarily on identifying oncogenes and tumor suppressors that initiate and promote the cancer phenotype (Bishop and Weinberg, 1996). Thus, the loss or gain of function in tumor suppressors or oncogenes induces changes in the regulatory mechanisms that control key processes associated with carcinogenesis, such as cell transformation, proliferation, invasion, intravasation, and metastasis (Thiery, 2002). Notably, several processes associated with the progress of this disease have been identified as the hallmarks of cancer. Currently, these hallmarks are sustained proliferative signaling, the evasion of growth suppressors, resistance to cell death, the induction of angiogenesis, the activation of invasion and metastasis, replicative immortality, tumor-promoting inflammation and the reprogramming of energy metabolism (Hanahan and Weinberg, 2011). With the recent advances in metabolome technology, there is now great interest in elucidating how metabolic profiles change to meet the metabolic demand during carcinogenesis. One of the most clear lines of evidence supporting these metabolic alterations in cancer cells is that cancer cells preferentially use glycolysis instead of aerobic respiration to obtain ATP even under aerobic conditions; this finding is known as the Warburg effect (Ward and Thompson, 2012). From an energetic point of view, glycolysis is a less efficient mechanism to produce ATP compared with oxidative phosphorylation (2 ATP molecules and 36 ATP molecules, respectively, produced from 1 molecule of glucose). Given the much higher efficiency of oxidative phosphorylation, a question arises: what is the metabolic advantage of using glycolysis to sustain the uncontrolled proliferation of cancer cells. To explain this apparent contradiction, Warburg proposed that cancer cells do not have functional mitochondria (Warburg, 1956); however, contrary to this hypothesis, there is evidence that the oxidative phosphorylation pathway is active in some human cancer cells (Weinhouse, 1976). Hence, the central question still requires an answer. To this end, there have been many hypotheses to explain this metabolic preference, for example, the hypothesis that the use of glycolysis is an effect of oxygen deprivation in the tumor. Oxygen deprivation is observed in some areas of solid tumors, and as a consequence, there could be reduced dependence of energetic metabolism on aerobic respiration (Braun et al., 2001). Furthermore, there is evidence that the prolonged exposure of cells to hypoxia promotes activity that favors the cancer phenotype, including the activation of hypoxia-inducible factor (HIF-1α), which is an important regulator of the metabolic activity of glycolysis and the glucose uptake rate (Robey et al., 2005; Brahimi-Horn et al., 2007). However, even though the activation of HIF-1α may be a reasonable explanation, HIF-1α has been shown to be expressed in tumor cells even in the presence of oxygen (pseudo-hypoxia), and therefore, this piece of the puzzle remains unsolved (King et al., 2006). In our opinion, finding the answer requires the combined use of high-throughput experiments and computational models able to determine which metabolic pathways are necessary to support the cancer phenotype and how these pathways are organized to support aerobic glycolysis during cancer cell proliferation. The understanding of such a phenomenon is crucial to the development of systematic schemes that contribute to identifying mechanisms that may reverse or delay the development of a malignant state. To contribute to this field of research, we present and discuss a computational analysis of the central metabolic pathway in cancer cell lines. This formalism—based on constraint-based modeling—allows us to (1) integrate high-throughput data, (2) generate biological hypothesis regarding the crucial pathways involved in cancer, and (3) design experiments to evaluate the *in silico* predictions. By analyzing specific examples, we provide evidence that this formalism can serve as a rational guide for identifying enzymatic targets with the potential to inhibit the cancer phenotype.

## High-throughput technology: top–down description

Integrative approaches in systems biology can be used to organize and interpret experimental data and to provide a greater understanding of the metabolic principles that underlie the cancer phenotype. To this end, high-throughput technologies (HTs) are a valuable tool to characterize the global activity of living organisms through the analysis of massive amounts of data on gene expression, protein concentrations, or metabolic profiles, to name a few examples. Importantly, the profiles obtained from these data constitute a way to characterize the phenotype of a microorganism through qualitative and quantitative procedures, both of which are important tools to assess the results obtained from computational models.

Overall, these technologies have contributed to the understanding of some mechanisms that trigger the cancer phenotype at diverse biological levels, and currently, there is an overwhelming number of genes, proteins, and metabolites whose activities are known to be associated with the evolution of this disease. For instance, Kreig et al. demonstrated in 2004 that alterations at the subunit level of a single enzyme complex (cytochrome c oxidase) are correlated with altered metabolism in tumors (Krieg et al., 2004).

In 2009, Sreekumar et al. reported the profiles of more than 1126 metabolites across 262 clinical samples related to prostate cancer. These unbiased metabolome profiles were able to distinguish benign clinically localized prostate cancer and metastatic disease (Sreekumar et al., 2009). Furthermore, Fan et al. studied the metabolic perturbations arising from malignant transformation in human lung cancers *in situ* (Fan et al., 2009). They investigated these metabolic changes by infusing uniformly labeled 13C-glucose into human lung cancer patients, followed by resecting and processing paired non-cancerous lung tissues and non-small cell carcinoma tissues, as well as blood plasma. Complementary, in 2010, Bottomly et al. used massively parallel sequencing (ChIP-seq) to provide evidence that the Wnt/β-catenin and mitogen signaling pathways intersect directly to regulate a defined set of target genes in colon cancer (Bottomly et al., 2010). Equally important, in 2010, Huarte and Rinn, using ChIP-seq, provided data that improved the understanding of the role that large ncRNAs have in cancer pathways (Huarte and Rinn, 2010). Large ncRNAs are also emerging as important regulatory molecules in tumor-suppressor and oncogenic pathways. Notably, the metabolic pathways associated with the cancer phenotype have been studied using these and others methods, and the potential control of metabolism has opened up an alternative avenue for designing novel therapeutic approaches in cancer treatment (Godinot et al., 2007). In 2009, Vanableset et al. published a study in which microarrays were used to show that approximately half of all active alternative splicing events in ovarian and breast tissues were altered in tumors, and many of these events seem to be regulated by the binding of a single factor: the RNA binding protein FOX2 (Venables et al., 2009). As we said before, there is an overwhelming number of examples showing the use of HTs to provide a greater understanding of cancer phenotype, but an extensive discussion of these achievements falls outside to the purpose of this review.

In light of these and other findings reported in the literature, carcinogenesis can be characterized as a particular metabolic phenotype in an organism that results from anomalous interactions among many biological components (genes, proteins, ncRNA, metabolites, etc.); in other words, carcinogenesis can be characterized as a dysfunctional state of a biological network. Thus, to understand the biochemical mechanisms that sustain cancer, it makes sense to study this disease in a collective way, i.e., as a result of interacting networks. At this stage, there are some issues that must be addressed; for instance, once we have experimental data regarding gene expression or the proteome, how do we handle these data and construct a coherent history of the metabolic activity associated with the cancer phenotype in a microorganism. Which metabolic pathways have a central role in cancer and how can these pathways be used to identify potential targets for clinical treatment. Despite the enormous contribution of HTs to the identification of the genetic and metabolic mechanisms that favor the development of cancer, most of these top-down schemes are descriptive and non-predictive. Thus, to move toward a quantitative and predictive scheme, the use of computational modeling for genome-scale metabolic reconstruction is necessary. In this context, there are different schemes for the computational modeling of metabolic pathways, but herein we focus on Flux Balance Analysis (FBA), a paradigm in systems biology whose utility to understand and predict metabolic states has been proven in a variety of microorganisms (Resendis-Antonio et al., 2007, 2010, 2011, 2012; Orth et al., 2010; Schellenberger et al., 2011; Benedict et al., 2012; Kim and Reed, 2012; Martinez et al., 2012; Paglia et al., 2012; Pardelha et al., 2012; Tilghman et al., 2012).

## Flux balance analysis

**Figure 1:**
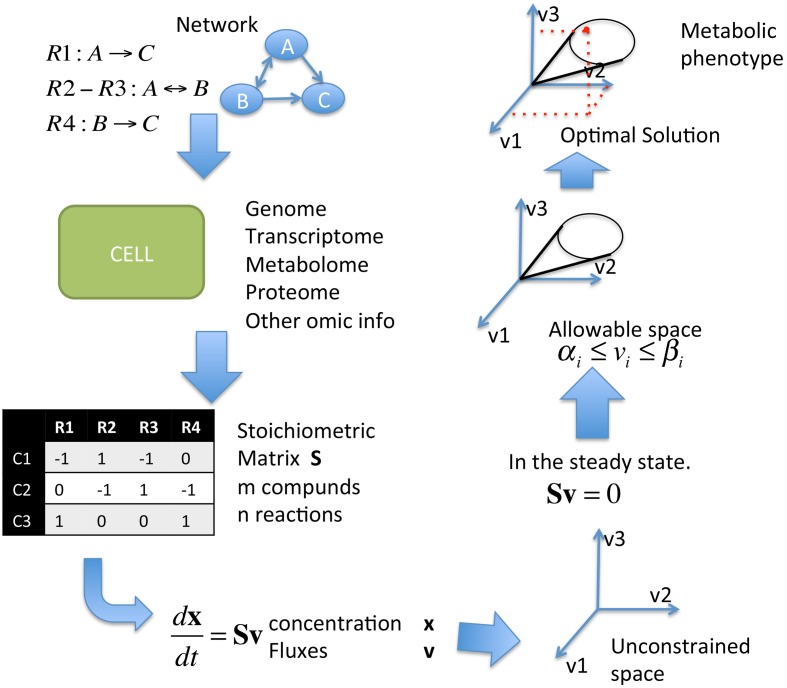
**Scheme of Flux Balance Analysis (FBA).** First is the definition of the network, HT data is used to complete different levels of information (either in the network, concentration, or fluxes), the network get represented in an stoichiometric matrix. Given the fluxes, the concentration of each metabolite and the stoichiometric matrix the steady state is found.

Constraint-based modeling is a successful framework in systems biology that has proven useful to explore the metabolic activity in microorganisms and whose principles and methods can be reviewed elsewhere. Among the computational methods classified in this framework, FBA constitutes a fundamental tool for integrating high-throughput data and assessing predictions regarding genotype–phenotype relationships (Orth et al., 2010; Schellenberger et al., 2011). Briefly, having selected the metabolic reconstruction of a microorganism, FBA lets us identify the space spanned by feasible metabolic capacities for the organism under steady state conditions by imposing thermodynamic, enzymatic, and regulatory constraints. This space contains an overwhelming number of metabolic states, and in turn each state represents different strategies that balance the metabolic requirements of the cell and the nutrients available in the environment. Importantly, not all these states produce equivalent metabolic phenotypes, for instance, an equivalent level of biomass production, because some states are more efficient than others. Thus, having selected a specific biological objective, for instance, biomass production, the problem in FBA is reduced to finding the metabolic state, or states, that maximize or minimize that phenotype. The elucidation of the metabolic state associated with a specific physiological situation requires two steps: (1) the reconstruction of an objective function (OF) that represents a biological process, which, for instance can be biomass production or the production of a specific metabolite; and (2) the identification of a metabolic state that maximizes or minimizes this state by an OF. A FBA workflow with the required data and methodologies is shown in Figure 1 and Appendix. Understanding the metabolic alterations that distinguish cancer cells from normal cells requires systemic and systematic schemes to uncover the mechanisms that support the cancer phenotype, and this understanding will facilitate the design of more effective treatments. In the remainder of this short review, we will specially focus on how FBA can be a useful tool for this latter purpose. Thus, the purpose of this paper is to show how *in silico* analysis can serve as a guide for modeling and exploring the metabolic consequences of the Warburg effect, the implications of this effect regarding the cancer phenotype and how genome-scale modeling can be used to identify enzymatic targets with potential therapeutic applications (Resendis-Antonio et al., 2010).

## Modeling cancer metabolism

**Figure 2:**
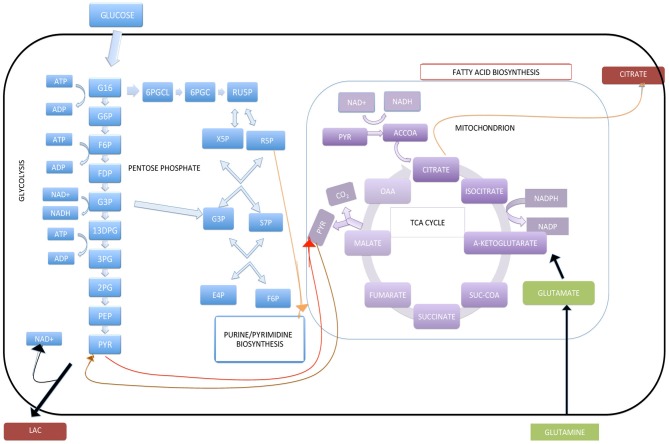
**Central metabolism in cancer cell lines.** LAC represents lactate, G6P represents glucose-6phosphate, F6P represents fructose 6 phosphate, FDP represents fructose 1,6 biphospate, G3P represents glyceraldehydes 3 phosphate, 13DPG represents 1,3 biphosoglycerate, 3PG represent 3 phosphoglycerate, 2PG represents 2-phopho glycerate, PEP represents phosphenol pyruvate, PYR represents pyruvate, 6PGCL represents 6-phosphoglucono-δ-lactone, 6PGC represents 6-phosphogluconate, RU5Prepresents ribulose 5-phosphate, R5P represents Ribose 5 phosphate, X5P represents Xylulose 5 phosphate, S7P represents sedoheptulose 7-phosphate, E4P represents erythrose 4-phosphate, OAA represents oxaloacetate, SUC-COA represents succinyl-CoA, ACCOA represents acetyl CoA.

As we described above, the Warburg effect is a fundamental metabolic process that contributes to the malignant transformation of most cancer cells. The fundamental nature of this effect makes it attractive to study, and consequently, the understanding of this effect could have conceptual and practical implications for new clinical treatments. With this aim in mind, in this section, we will briefly discuss how constraint-based modeling can be used as a computational tool to characterize the metabolic activity of cancer cells during aerobic glycolysis. Furthermore, we will focus on showing the practical implications of constraint-based modeling that may interest biomedical researchers in the field of cancer research. For the sake of simplicity, our results are constrained to a metabolic reconstruction whose set of reactions represents the central metabolism in a cell exposed to specific external conditions (see Figure 2). Although this metabolic network—containing only 89 reactions and 98 metabolites—is only a subset of the human metabolic reconstruction, we argue that it serves as a baseline to exemplify the method and, simultaneously, as a benchmark to assess the *in silico* predictions in terms of the experimental data currently found in the literature. In particular, we will show that our method can be used as an auxiliary computational tool for identifying a set of enzymes with an important role in supporting the metabolic phenotype in cancer cells. To explore the metabolic activity in cancer cells using the perspective of constraint-based modeling, three requirements should be met systematically: (1) the metabolic reconstruction of a human cell, (2) the definition of an OF associated with a physiological state, and (3) the completion of computational simulations and the experimental assessment of their outputs.

## Metabolic reconstruction

Once one has identified the organism of study, the immediate questions are how to define the physical limits of the system and how to define the number of metabolic reactions in the network to be included in the analysis. With the goal of analyzing the metabolic state associated with the Warburg effect, we assumed that the following metabolic pathways define our system: glycolysis, the pentose phosphate pathway, the TCA cycle, and oxidative phosphorylation (see Figure 2). In addition, we considered our system to be capable of importing glucose and glutamine and exporting lactate. These properties are in agreement with the fact that cancer cells have an increased uptake rate of glucose and that carcinogenesis proceeds in an acidic microenvironment.

In the rest of this work, our discussion will be centered on a metabolic reconstruction of the central metabolism in human cells. As we show, this metabolic reconstruction and its computational modeling have two advantages: (1) this model can demonstrate how to explore the relationship between constraint-based modeling and phenotype in cancer cell lines, and (2) this model constitutes a proper framework to explore how the perturbations of some enzymes participating in central metabolism can affect the cancer phenotype. Clearly, this latter information is necessary to calibrate the model and evaluate the *in silico* predictions. However, given that metabolic alterations in cancer cells have consequences with respect to network organization, there will be additional pathways—not considered herein—that can play an important role in driving carcinogenesis. This latter point is an avenue that should be explored in more detail. Despite this simplification, we show that this first approach meets the goals of this paper: to provide evidence that constraint-based modeling is a coherent framework to analyze the metabolic state associated with the cancer phenotype and to predict the genotype–phenotype relationship in cancer cell lines.

## Biomass production by cancer cells: construction of a proper objective function

As we explained in the previous sections, an OF is required to perform FBA and find the metabolic state associated with specific environmental conditions. From a phenomenological point of view, cancer cells exhibit a metabolic alteration that ensures the biosynthesis of the building blocks required to meet the demand generated by uncontrolled cell proliferation. To identify this dysfunctional metabolic state, we constructed an OF by selecting those metabolites in our reconstruction that are utilized as precursors for producing amino acids, nucleotides, fatty acids, electron carriers, and compounds involved in energy transfer. Hence, taking into account this argument, the OF can be written as:

$$\text{OF}\left(\text{Biomass}\right)={\text{c}}_{\text{1}}\text{ATP}+{\text{c}}_{\text{2}}\text{NADH}+{\text{c}}_{\text{3}}\text{NADPH}+{\text{c}}_{\text{4}}\text{R5P}+{\text{c}}_{\text{5}}\text{ATP}+{\text{c}}_{\text{6}}\text{SUCC}$$

where all the coefficients *c*_*i*_, *i* = 1 … 6, represent the weight factors required to produce one unit of biomass (nmol/gDW· hr^−1^). In this context, it is necessary to identify the metabolic profile that optimizes the OF, and these conditions represent those that yield the maximal biomass production associated with the proliferation of cancer cells. It is important to mention that given that the demand for the production of these metabolites is equally required during normal cell proliferation, our main hypothesis to distinguish the metabolic state associated with normal proliferation from the metabolic state associated with cancer is to assume that this latter state requires a metabolic profile that maximizes the OF. In other words, given that the cancer phenotype is related to uncontrolled cell proliferation, it is a plausible hypothesis that the relationship among the metabolic pathways that maximizes the production of biomass is closely related to cancer metabolism.

## Exploring the genotype–phenotype relationship in cancer metabolism

**Figure 3:**
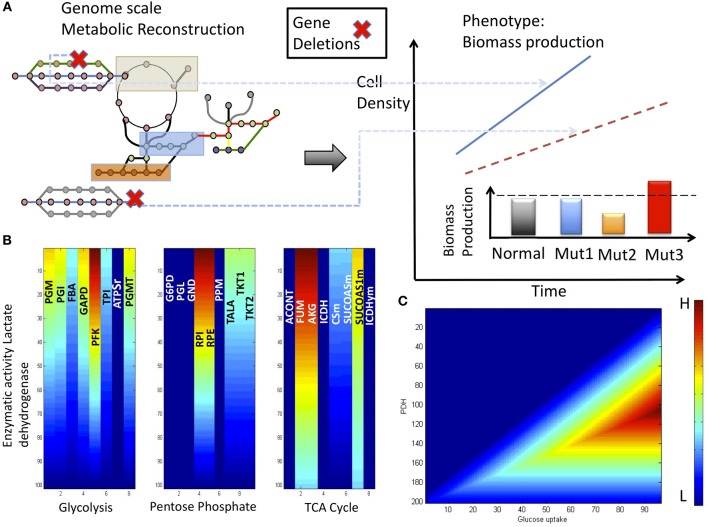
**Analysis with constraint-based modeling. (A)** Having reconstructed the metabolic network, *in silico* gene deletion allows to identify the phenotype behavior in cancer cell lines. In this case, phenotype is defined in terms of biomass production. Three effects can occur in this situation: (1) genes whose activity is dispensable, (2) genes whose expression reduce the biomass, and (3) genes whose activity is essential to biomass production. Based on *in silico* analysis, we conclude that lactate dehydrogenase (*LDH*) has a pivotal metabolic control on cancer cell growth (Resendis-Antonio et al., 2010). Supporting this finding, panel **(B)** shows the effects that variations on the enzymatic activity of *LDH* have on some enzymes participating in glycolysis, TCA cycle and pentose phosphate. As the figure shows according the metabolic activity of *LDH* decrease, we note a reduced activity on all the enzymes integrating these pathways. Regions in red and blue represent a higher (H) and lower (L) metabolic flux activity, respectively. **(C)** Phenotype phase plane considering the activity of pyruvate dehydrogenase (*PDH*) and glucose uptake rate.

Having defined the uncontrolled cell proliferation in cancer cells using the OF, we can characterize the feasible space of metabolic responses using FBA and eventually identify and explore those metabolic pathways that support the cancer phenotype. Hence, under this assumption, we can identify the metabolic state or states that maximize the OF and, in turn, elucidate the entire metabolic mechanism required to support the cancer phenotype (see Figure 3). Some advances have been reported recently in this type of scheme for studying metabolism in cancer cells. For instance, it has been reported that the constraint-based modeling of central metabolism was able to qualitatively reproduce an experimental growth curve for the HeLa cancer cell line (Resendis-Antonio et al., 2010). Notably, this study provides a computational methodology that contributes to identifying the metabolic reactions whose activities could be crucial to support the cancer phenotype. This latter issue is a worthy goal to optimize the design of drugs targeting human cancer (see Figure 3).

Among the target enzymes identified by this *in silico* analysis are glucose transporters and several glycolytic enzymes that are essential for optimizing biomass production enolase (*ENO*), glyceraldehyde-3-phosphate dehydrogenase (*GAPD*), phosphoglycerate (*PGC*), pyruvate kinase (*PYK*), and lactate dehydrogenase (*LDH*) (Resendis-Antonio et al., 2010). Consistent with these results, targeting glucose transporters and the phosphorylation steps in glycolytic pathways has been shown to be a complementary strategy for inhibiting the cancer phenotype (Gatenby and Gillies, 2004; Xu et al., 2005; Christofk et al., 2008). For the sake of illustration, in the last part of this article, we will analyze two specific enzymes identified in this analysis: *LDH* and *pyruvate dehydrogenase*. An extensive analysis that includes other metabolic pathways has been reviewed previously (Resendis-Antonio et al., 2010). Both enzymes were identified through constraint-based modeling, and in agreement with this result, there is experimental evidence suggesting that the control of the activities of these enzymes can be used as a strategy to regulate the cancer phenotype (see Figure 3) (Shaw, 2006; Hsu and Sabatini, 2008).

LDH contributes to the acidification of the microenvironment and stimulates cancer cell proliferation via the transformation of pyruvate into lactate in the cytoplasm, which consequently is exported outside the cell. As Figure 3 shows, simulations with FBA under low oxygen uptake rates—emulating hypoxia—show that a decrease in the enzymatic activity of LDH is followed by a decreased metabolic activity in the form of glycolysis, as well as decreases in the rates of some reactions in the TCA cycle and pentose phosphate pathway (Resendis-Antonio et al., 2010). In the case of glycolysis, this metabolic effect has been observed experimentally, and the increased rate of lactate production has been proposed to be a necessary condition for the Warburg effect (Dang and Semenza, 1999). Even more important for practical purposes, there is experimental evidence that the inhibition of LDH reduces the activities of some glycolytic enzymes and consequently can be an alternative mechanism to reduce the growth rate in cancer cells (Fantin et al., 2006).

On the other hand, constraint-based modeling suggests that *pyruvate dehydrogenase (PDHm*) can perform a central role driving cell proliferation in cancer cell growth (see Figure 3). Supporting this computational finding, there is evidence that the metabolic inhibition of *PDHm* contributes to the Warburg effect, and in the case of human neck and head squamous carcinomas, the inhibition of *PDHm* enhances the malignant phenotype (McFate et al., 2008; Jones and Thompson, 2009). This observation makes sense given that the activation of HIF negatively regulates the catalytic activity of PDHm through the activation of *pyruvate dehydrogenase kinase 1* (see Resendis-Antonio et al., 2010 and the references therein). Simultaneously, the metabolic phenotype of cancer is favored because HIF induces the overproduction of enzymes that participate in the glycolytic pathway (Dang and Semenza, 1999). In summary, these *in silico* studies led us to conclude that the combined effect of an increase in aerobic glycolysis (the Warburg effect) and a decrease in the activity of *PDHm* seems to confer a selective advantage for survival and cell proliferation in cancer cells.

Another interesting question that we can address with constraint-based modeling is the effect of alterations of two selected metabolic reactions in the reconstruction on the cancer cell phenotype (Varma and Palsson, 1994; Price et al., 2004). For this purpose, phenotype phase plane (PPP) analysis is a simple computational procedure to visually explore the value of an OF as a function of alterations in two selected metabolic fluxes. For instance, PPP analysis based on the metabolic activity of PDH and glucose transporters has suggested that an increase in the glucose uptake rate combined with a decrease in the PDHm enzymatic activity may increase the growth rate of cancer cell lines only inside of certain region of the feasible metabolic space (see Figure 3). Thus, as one can imagine from these interpretations, constraint-based modeling contributes to the development of biological hypotheses and, in turn, the design of experiments to test these hypotheses.

In light of these results, constraint-based modeling represents an effort to construct a computational platform that can serve as a guide for the descriptive and predictive analysis of cancer metabolism involving continuous dialog with the experimental data.

## Conclusions

Cancer is a multifactorial disease, and the development of effective clinical treatments requires new paradigms and multidisciplinary strategies that systematically integrate (1) physiological knowledge, (2) experimental data on cell activity obtained using different HTs, and (3) an integrative description of these data in terms of computational modeling. In the case of human cancers, there are a variety of mechanisms by which this dysfunctional phenotype can be triggered; however, some hallmarks have been suggested to be independent of the type of cancer, and they are commonly present in all cancers (Hanahan and Weinberg, 2011). Among these hallmarks, the metabolic transformation that occurs during carcinogenesis is a promising marker that can be used to understand the progression of the disease and its principles and to potentially move toward optimal schemes in drug design. In this work, we have demonstrated how a computational model, based on constraint-based modeling, can be useful to explore the metabolic profile associated with the cancer phenotype. Furthermore, we have shown how this formalism can eventually be useful to identify proteins whose enzymatic activities could be essential to support the cancer phenotype.

Even though the *in silico* model involves numerous simplifications, we argue that this type of scheme can be used to explore fundamental and practical issues related to cancer in a systematic and systemic manner. Thus, the *in silico* identification of *lactate* and *pyruvate dehydrogenase* as two potential enzymes necessary to support the cancer phenotype exemplifies how constraint-based modeling in systems biology can be useful to understand how metabolism participates in carcinogenesis and, eventually, how such modeling could be used in the design of experimental strategies to limit or delay the progression of the disease. In this review, we have discussed only two enzymes obtained from the FBA, however, other results can be obtained and consequently require further analysis. For instance, constraint-based modeling has suggested that mitochondria-derived citrate constitutes a fundamental metabolite that supports cell proliferation. Thus, taking into account this information in the computational model, the inhibition of *ATP citrate lyase* in the cytoplasm prevents cancer cell proliferation and tumor growth due to its role in lipid biosynthesis (Hatzivassiliou et al., 2005; Hsu and Sabatini, 2008). Although a simple explanation of this effect is that the inhibition of *ATP citrate lyase* has the final effect of reducing the production of acetyl-coenzyme, a more detailed analysis should be performed in the future that considers the additional metabolic pathways that can contribute to the formation of acetyl-coenzyme A, for instance, fatty acid metabolism. Overall, these results provide evidence that FBA is an appropriate formalism to simultaneously advance three areas: (1) the integration of high-throughput data from cancer studies, (2) the construction of a formal platform to develop biological hypotheses, and (3) the design experiments to assess the genotype–phenotype relationships identified using *in silico* procedures.

In our opinion, this paradigm in systems biology—constraint-based modeling—constitutes a cornerstone for the construction of systemic models that will contribute to the understanding of the metabolic activity in cancer cells. Undoubtedly, advances in this understanding will be necessary to create a new paradigm in biomedical sciences to elucidate the changes in normal cell activity and improve the effectiveness of the clinical treatment of human cancers.

### Conflict of interest statement

The authors declare that the research was conducted in the absence of any commercial or financial relationships that could be construed as a potential conflict of interest.

## Appendix

The set of chemical reactions that comprises a network can be represented as a set of equations including stoichiometric information. This information can be represented in matrix form using the stoichiometric matrix, denoted by S. This structure represents the key for the *in silico* analysis. The stoichiometric matrix represents a linear transformation between the flux vector (**v**) and the concentration vector (**x**).

$$\begin{array}{l} \frac{dx}{dt}=S\cdot v\\ \;x=\left(\begin{array}{c} x_{1}\\ x_{2}\\ \cdot \\ \cdot \\ x_{m} \end{array}\right)\text{ and }v=\left(\begin{array}{c} v_{1}\\ v_{2}\\ \cdot \\ \cdot \\ v_{n} \end{array}\right) \end{array}$$

FBA is a computational framework for analyzing and exploring the phenotypic space of metabolic networks. FBA uses **S,** and obeys mass conservation and the steady state assumption (no variation in the concentration over time). For example, if we identify the metabolic fluxes that maximize biomass production when constrained by thermodynamics and mass balance principles, we have:

$$\begin{array}{l} \max\left[\text{biomass}=\sum_{i\;=\;1}c_{i}\cdot X_{i}\right]\\ \text{such that}\\ \sum_{S_{i,\;j}}\cdot \nu_{j}=0\;i=1,2\ldots m\\ -\alpha_{j}\leq \nu_{j}\leq \beta_{j}\;j=1,2\ldots n \end{array}$$

where, *S*_*ij*_, are associated with the *i-th* metabolite in the *j-th* reaction. Cellular growth (*X*_*i*_) is related to a combination of essential metabolites, whose contributions are weighted by the coefficients *c*_*i*_. In addition, the parameters α and β provide information about the limits of the enzyme capacities for each metabolic reaction.
